# Self-Swabbing for Virological Confirmation of Influenza-Like Illness Among an Internet-Based Cohort in the UK During the 2014-2015 Flu Season: Pilot Study

**DOI:** 10.2196/jmir.9084

**Published:** 2018-03-01

**Authors:** Clare Wenham, Eleanor R Gray, Candice E Keane, Matthew Donati, Daniela Paolotti, Richard Pebody, Ellen Fragaszy, Rachel A McKendry, W John Edmunds

**Affiliations:** ^1^ Department of Infectious Disease Epidemiology London School of Hygiene and Tropical Medicine London United Kingdom; ^2^ London Centre for Nanotechnology University College London London United Kingdom; ^3^ Division of Medicine University College London London United Kingdom; ^4^ Bristol Public Health Laboratory Public Health England Bristol United Kingdom; ^5^ ISI Foundation Torino Italy; ^6^ Centre of Infectious Disease Surveillance and Control Public Health England London United Kingdom; ^7^ Centre for Public Health Data Science Institute of Infectious Disease Informatics University College London London United Kingdom

**Keywords:** influenza, influenza-like illness, surveillance, online, cohort study, virological confirmation

## Abstract

**Background:**

Routine influenza surveillance, based on laboratory confirmation of viral infection, often fails to estimate the true burden of influenza-like illness (ILI) in the community because those with ILI often manage their own symptoms without visiting a health professional. Internet-based surveillance can complement this traditional surveillance by measuring symptoms and health behavior of a population with minimal time delay. Flusurvey, the UK’s largest crowd-sourced platform for surveillance of influenza, collects routine data on more than 6000 voluntary participants and offers real-time estimates of ILI circulation. However, one criticism of this method of surveillance is that it is only able to assess ILI, rather than virologically confirmed influenza.

**Objective:**

We designed a pilot study to see if it was feasible to ask individuals from the Flusurvey platform to perform a self-swabbing task and to assess whether they were able to collect samples with a suitable viral content to detect an influenza virus in the laboratory.

**Methods:**

Virological swabbing kits were sent to pilot study participants, who then monitored their ILI symptoms over the influenza season (2014-2015) through the Flusurvey platform. If they reported ILI, they were asked to undertake self-swabbing and return the swabs to a Public Health England laboratory for multiplex respiratory virus polymerase chain reaction testing.

**Results:**

A total of 700 swab kits were distributed at the start of the study; from these, 66 participants met the definition for ILI and were asked to return samples. In all, 51 samples were received in the laboratory, 18 of which tested positive for a viral cause of ILI (35%).

**Conclusions:**

This demonstrated proof of concept that it is possible to apply self-swabbing for virological laboratory testing to an online cohort study. This pilot does not have significant numbers to validate whether Flusurvey surveillance accurately reflects influenza infection in the community, but highlights that the methodology is feasible. Self-swabbing could be expanded to larger online surveillance activities, such as during the initial stages of a pandemic, to understand community transmission or to better assess interseasonal activity.

## Introduction

Influenza and influenza-like illness (ILI) cause a considerable burden of illness in the UK [1]. For most people, influenza is usually a self-limited disease for which, on average, between 1.5 and 4.9 working days are lost for each episode [2]. The consequences for high-risk groups (very young, older people, pregnant women, and those with an underlying health condition) can be more serious. Public Health England (PHE) estimate that each winter hundreds of thousands of people see their general practitioner (GP), tens of thousands are hospitalized, and there are on average 8000 deaths because of influenza [3-5]. Moreover, it has been estimated in 2011 that an outbreak of pandemic influenza could reduce the UK gross domestic product by approximately 1.14% to 1.42% (£14.8-£18.5 billion) [6].

Surveillance is an essential function for monitoring seasonal and pandemic influenza, delivering epidemiological, virological, and clinical awareness of the circulating virus and studying interventions, such as vaccination programs [7]. However, routine surveillance through medical settings (GPs and hospitals), based on laboratory confirmation of infection, does not provide a full picture of the true societal burden of influenza at any one time due to the fact that individuals suffering from ILI often do not visit a health care professional, but manage their symptoms on their own [8,9]. Syndromic surveillance of ILI is increasingly used as a method for detecting discernible trends in illness, without laboratory confirmation [10]. For example, PHE collates data from a range of sources to compile its weekly national influenza reports, including NHS 111 (a nonemergency health advice phone line), the Royal College of General Practitioners’ (RCGP) Weekly Returns Service (GP-based sentinel surveillance), Medical Offices of Schools Association, community telephone surveys, and online disease surveillance platforms such as Flusurvey [11].

Flusurvey, the UK’s largest crowd-sourced platform for surveillance of influenza, collects routine data on more than 6000 voluntary participants [12]. On registration, a baseline epidemiological questionnaire is carried out, asking about individuals’ age, gender, location (first part of postcode), household composition, influenza vaccine status, and preexisting health conditions. Although Flusurvey participants are not representative of the UK population, adjustments are made through modeling processes to allow for broader calculations to be made at the population level. Subsequently, participants are emailed each week to complete a symptoms survey. Participants select recent symptoms from a list including respiratory and gastrointestinal concerns, and provide information relating to onset and duration of symptoms and health-seeking behavior, as well as rating how they are feeling overall on a scale of 1 (very unwell) to 100 (in excellent health) [13]. Accordingly, by gathering these datasets, it is possible to estimate, with minimum delay, the incidence of ILI among Flusurvey participants, which has been previously shown to correlate with the incidence measured by sentinel-based surveillance at PHE [14].

Internet surveillance can complement traditional surveillance by measuring symptoms among a population with minimal time delay [15]. When this is done continually, it can improve the quality of the incidence data and help to inform policy decisions during routine seasonal influenza and pandemics [15]. The use of internet-based disease surveillance has increased rapidly in the past decade, including online ILI syndromic survey systems, such as the Europe-wide Influenzanet (of which Flusurvey is a member, working in synergy with other European platforms sharing data collection modality and results) [16], FluNearYou in the United States [17], and Australia’s Flutracking [18]. Estimates of the relative incidence of influenza have also been inferred from search engine query data [19], detecting news reports from news sites aggregators [20], social media platforms such as Twitter [21], Wikipedia access logs [22], restaurant reservation and review logs [23], nonprescription pharmacy sales [24], and prediction markets [25]. Moreover, online crowd-sourced surveillance platforms have been similarly developed for other health conditions, including malaria [26], food-borne illness [27], and tick-borne diseases such as Lyme disease [28].

One criticism of online surveillance for influenza (and other syndromic surveillance mechanisms) is that because data are based on self-reporting of symptoms, the results collected are only representative of ILI, rather than virologically confirmed influenza [29]. Although previous years of ILI incidence from Flusurvey corresponded to RCGP influenza data, suggesting that Flusurvey does detect outbreaks of influenza, this has not been confirmed. Virological confirmation studies are required to assess how the measured ILI rates compare with the actual circulation of influenza in the population. Previously, two studies have sought to do this: one undertook a virological self-sampling from those calling the UK national telephone health helpline (NHS Direct) [30,31] and one completed virological self-sampling of GoViral participants in Massachusetts [32]. Similar self-testing or self-sampling studies have been undertaken for HIV/AIDS [33-35] and chlamydia, where the online cohort approach has been combined with a complete eSexual Health Clinic [36].

We designed a pilot study to assess whether it was feasible to detect laboratory-confirmed influenza from an online cohort. We monitored ILI through the Flusurvey platform, asking those reporting ILI to undertake a nasal swab for laboratory assessment of whether they were actually infected with an influenza virus, another respiratory virus, or unknown etiology (including viruses not tested for). This pilot study will help to assess the validity of online platforms for ILI surveillance and to confirm if the syndromic cohort surveillance approach is able to detect influenza infection. Moreover, it will additionally serve as a proof-of-concept study, showing that self-testing in the community can be successfully added to internet-based surveillance of ILI.

## Methods

We recruited unvaccinated volunteer participants by including “Would you be interested in taking part in a virological self-swabbing study?” on Flusurvey’s regular baseline recruitment survey [12]. Only unvaccinated participants were selected to offer increased chances of testing positive for influenza. Recruitment into the pilot was open from November 17, 2014 to December 17, 2014, with 1615 potential participants volunteering. As a feasibility study, limited by financial constraints, we sought to generate 100 swabs for testing. Based on experience from Flusurvey and the Flu Watch study [9], we estimated that approximately 48% of participants would experience a respiratory illness during periods of influenza circulation, 43% (3/7) of those would have an onset on the day or within the 2 days of reporting the illness (important for a high viral load to facilitate laboratory testing), and that 70% of those requested to return a swab would do so. Given these parameters a total of 700 individuals were recruited to take part in the self-swabbing pilot. Accordingly, we purposefully sampled 700 of 1615 eligible participants to include high-risk groups for influenza infection, including all those younger than 18 years and older than 65 years who volunteered or were volunteered by parents [37,38]. Random sampling of those aged between 18 and 64 years was then undertaken to reach a total of 700 participants. We sent these 700 participants a cover letter, an information sheet on the project, the virological swabbing kit, and sample transport materials (Multimedia Appendix 1). The virological swab kit contained a regular tip flocked swab plus 1 mL Universal Transport Medium (Sterilab, North Yorkshire, UK). For transportation of the sample to the laboratory, the pack contained a round mailing container (126 × 30 mm) with liner, a neutral screw cap for the mailing container, and a mailing box packaging system for containers up to 30 mm (Sarstedt, Numbrecht, Germany) to meet Category B UN3373 posting standards for viral materials. Also included were instructions for undertaking the swabbing (Multimedia Appendix 2), a link to video instructions [12], and identification labels for the samples (Multimedia Appendix 3). Participants were asked to store it safely until instructed to self-swab. Informed consent was obtained from pilot study participants when they selected that that they were willing to take part in the study online.

Participants were asked to report their symptoms online on a weekly basis. If their symptoms met the European Centre on Disease Prevention and Control definition of ILI, which is sudden onset of symptoms and at least one of four systemic symptoms (fever or feverishness, malaise, headache, myalgia) and at least one of three respiratory symptoms (cough, sore throat, shortness of breath) [39], and if the reported date of onset of symptoms was within 4 days of notification (to ensure virological testing during the acute phase of infection with expected higher viral loads [40]), then participants were asked to self-swab. Due to low circulation of influenza during the time period of the pilot study [14], the swabbing criteria were expanded on March 16, 2015, to include acute respiratory infection (ARI). (This comprises the sudden onset of symptoms and at least one of the following four respiratory symptoms: cough, sore throat, shortness of breath, and coryza [39].)

Nasal swabbing was chosen because it has been shown to be effective for influenza testing by patients [41] and offers a greater viral load than saliva collection [32]. Although nasopharyngeal swabbing may yield a slightly greater viral load for confirmation with polymerase chain reaction (PCR) tests [42], we believed it would be difficult to self-administer nasopharyngeal swabs of consistent quality and therefore flocked nasal swabs provided a suitable alternative [43]. Participants were provided with both written and video instructions as to how to administer the self-swab (Multimedia Appendix 2).

Participants were asked to send their samples (Multimedia Appendix 2) to the PHE Laboratory, Bristol, for respiratory virus PCR testing. The samples were assayed using PHE Bristol in-house–validated PCR panels including targets for common respiratory infections, including influenza A, influenza B, respiratory syncytial virus (A and B) human metapneumovirus, parainfluenza virus (1, 2, and 3), adenovirus, and rhinovirus. This assay was chosen based on its confidence in detecting circulating strains of influenza. The PHE Laboratory used a generic influenza A assay that is assessed *in silico* against common circulating strains (primer and probe matching) and then tested in practice using a proficiency panel constructed by the respiratory virus unit from PHE, plus other external quality assurance schemes. Results were returned to researchers at the London School of Hygiene and Tropical Medicine and sent to participants via email. As this was a pilot research study, rather than a clinical test, participants were informed before self-sampling that their results would not be available in real time.

It was important to ascertain whether samples with negative results through the PCR multiplex testing were truly negative or whether the test had not been administered properly, providing a false negative. Second-stage testing was undertaken at London Centre for Nanotechnology, University College London. These samples were quantified for human nuclear and mitochondrial DNA by TaqMan PCR for mammalian glyceraldehyde 3-phosphate dehydrogenase (GAPDH) DNA in the swab samples [44]. Positive controls for the reaction were either HeLa cell DNA (NEB, Hitchin, UK) or human placental DNA (Sigma, Dorset, UK). Cycling conditions were 95°C for 15 seconds and annealing/extension at 60°C for 1 minute after an initial denaturation of 10 minutes.

Finally, we asked those participants to complete a short evaluation form online to ascertain how easy they found the process and its viability for future (Multimedia Appendix 4).

Ethics approval for undertaking this study was obtained from Observational and Interventions Research Ethics Committee at London School of Hygiene and Tropical Medicine (ref: 5530-03).

## Results

Samples were received at PHE Laboratory, Bristol, from January 1, 2015 to April 7, 2015. In total, 66 participants from the pilot group (of the 100 originally estimated based on sample size calculations) met the symptom and timing of onset criteria and were asked to self-swab. A total of 51 swab samples (77%) were received at PHE Laboratory. An additional three samples were received, although they were not requested, and therefore they were not included in the analysis. Multiplex PCR testing results are presented in Table 1 and Multimedia Appendix 5.

The second-stage testing for the presence of human DNA produced the results presented in Table 2 and Multimedia Appendix 6.

These findings show all samples contained human DNA and are consistent with the correct use of the swab, corroborating earlier successful experiences of self-swabbing in a home setting [9,32,41]. This validated the feasibility and the process used as well as the respiratory virus detection results.

Reflecting similar demographic trends from the broader Flusurvey project [45], including from the 2014-2015 cohort from which these participants were selected [46], the swabs received at the laboratory for virological testing were not representative of the UK population. Of the 51 results received, 36 were received from female participants and 15 were from male participants. The age of participants ranged from 4 to 91 years with a mean age of 41 (SD 19) years.

As part of the routine information collected by Flusurvey, participants are asked to score how they were feeling each week [13]. Over the course of the 2014-2015 influenza season, the mean reported score for all Flusurvey users (N=6102) was 82.9. This is self-reported on a scale from 0 to 100. For the 66 self-swab participants, the mean score for the weeks they reported ILI (or ILI and ARI after March 16, 2015) was 63.9 (SD 23.9). From those who tested positive for ILI, the range of scores was 30 to 100 and the mean was 72.8 (SD 16.9). Finally, for those who tested positive for influenza, the range was 60 to 85 and the mean was 72 (SD 9). Although there is a great risk of overinterpretation with a small sample size, and a risk of bias by characteristics of people self-swabbing, these results do not suggest a positive relationship between incidence of ILI and (self-reported) severity of symptoms through the health score.

Completed evaluation forms were received from 21 participants. Of these, 20 participants suggested that undertaking the swab was easy or very easy, although one stated it was “unpleasant.” In addition, 13 participants indicated that if they were to have ILI symptoms in the future, they would prefer to undertake a self-swab at home to diagnose symptoms, five participants preferred to treat symptoms at home without a swab, and three participants were undecided about their future use of swabbing at home. All those who completed the evaluation form found the written instructions helpful, and 13 participants found the video instructions useful, with the remaining eight participants not watching it.

**Table 1:** Yield of influenza-like illness positive tests from samples tested (N=51) using multiplex quantitative PCR..

Virus	Positive tests, n (%)
Influenza A (H3N2)	1
Influenza B	4
Human metapneumovirus^a^	2
Rhinovirus^a^	11
Inhibitory samples^b^	2
Total viral yield	18 (35)
Total influenza yield	5 (10)

^a^One sample tested positive for both human metapneumovirus and rhinovirus. We have included both of these infections separately in this table, but this reflects one dual infection.

^b^Two samples contained inhibitory substances that hindered amplification of sample control markers. No conclusions can therefore be drawn from these samples, positive or negative.

**Table 2:** Detection of mammalian DNA using Taqman quantitative polymerase chain reaction.

Group	Samples, n	GAPDH level (Ct^a^), mean (SD)
No virus detected	20	28.4 (2.9)
Virus detected	11	28.7 (2.5)
Low virus detected	5	28.2 (4.7)
Inhibitors present	2	26.1 (3.7)
Not tested for virus	3	23.7 (1.6)

^a^Ct: cycle threshold. A Ct value <40 represents positive detection of human DNA.

## Discussion

This study has shown that, as a proof of concept, it is possible to successfully apply an at-home self-swabbing methodology to an internet-based cohort and that this can detect both influenza and other causes of ILI by collecting viral samples of suitable quality for PCR multiplex testing. This replicated findings of similar studies conducted for self-swabbing from an online cohort through the GoViral Platform in the United States, through phone-based surveillance via NHS Direct, and in community self-swabbing through Flu Watch [9,31,32], extending these to assess feasibility among a crowd-sourced platform. It was estimated that in the 2014-2015 influenza season, there were low to moderate levels of influenza activity with the predominant strain influenza A (H3N2) [14] present for the majority of the season (the majority were antigenically similar to the A/Texas/50/2012 H3N2 Northern Hemisphere strain), and the appearance of influenza B during the last months of the season (the majority of these belonged to the B/Yamagata 16/88 lineage) [14]. This pilot study did not generate a large yield of ILI virus-positive results, nor was it powered to, yet there were similar trends between this pilot and PHE Laboratory–confirmed cases for the same time period and in particular from the RCGP sentinel swabbing scheme, to which this would be most similar for detecting influenza in a community cohort [14].

Participants found the self-swabbing easy to undertake and several individuals in the pilot indicated that they would be interested in using a similar self-swab in the future if they wanted laboratory confirmation of ILI infection. This was a research project, therefore testing of the samples was not undertaken in real time and results were not returned to participants for a number of weeks, by which time their symptoms would have likely subsided, meaning that the results of the virological test would not have affected their behavior. However, if self-swabbing were to be conducted and results returned in real time, the results may affect patient behavior, either through visiting a health professional, taking medication, or changing their daily routine to limit potential viral spread. Moreover, virological confirmation of a viral infection may potentially reduce antibiotic prescription due to misdiagnosis.

### Usability of Findings

Yet, we do not wish to suggest that this methodology should replace the efficient RCGP sentinel swabbing system; our approach may not be practical for routine influenza surveillance owing to the cost and logistics of distributing kits. However, self-swabbing of an internet-based cohort may prove useful for ad hoc surveys, such as in the emerging stages of a pandemic to understand community transmission or as a supplementary tool to otherwise established surveillance mechanisms. Our study can also contribute to demonstrating the feasibility of both an online cohort approach to surveillance and self-swabbing at home for other health conditions, such as sexually transmitted infections or gastroenteritis for which there might be privacy reasons for patients seeking to test themselves at home.

Although this pilot study cannot make conclusive remarks about the validity of online influenza surveillance, it has shown that, as a proof of concept, it is possible to detect an influenza virus and other ILI from a cohort of online participants. Accordingly, this can be replicated at larger scale for greater verification of online crowd-sourced disease surveillance mechanisms. Moreover, if a self-swabbing study were to be repeated with a greater number of participants and samples, from a more representative demographic sample, it may be possible to build these into a strong analytical model for estimating the burden of influenza.

### Limitations

Due to delays in procuring the necessary materials, delays in obtaining ethical approval, postal delays, and closures due to Christmas, the self-swab kits were not distributed to participants until the first week of January 2015. Retrospectively, it can be seen that the peak of the influenza season in the UK during the 2014-2015 season was week 52 (December 22-28, 2014) [14]. As such, the study did not take place during the peak influenza season in the UK and there were not high levels of influenza circulating during the pilot period. Accordingly, we did not have a large sample group of swabs nor did we obtain the predicted 100 swabs. Despite the methodological change to include ARI on March 16, 2015 [39], this still failed to collect the original requirement of 100 samples. This trend matched PHE’s microbiological surveillance for the same time period, with a similarly low yield of influenza-positive samples [14]. However, the pilot study did coincide with the later peak of influenza B [14], and hence detections of influenza B.

If this project were to be undertaken again, swab kits should be distributed to participants before the start of the influenza season to mitigate the uncertainty of predicting when the virus may arrive and/or be at its peak. Alternatively, kits should be sent out, or be collectable locally, after notification of relevant symptoms. Although this delay may impact the viral load collected, it would prove more cost effective in a health care setting.

The participants who sent samples to the laboratory for testing were not a representative sample demographically, featuring predominantly women aged between 18 and 64 years in Southeast England. This reflects Flusurvey and Influenzanet participants more generally and does not represent a random sample of the UK/European populations [47]. Any future self-swabbing studies carried out on an internet-based cohort could broaden the demographics of the sample to increase the study’s applicability. This could include greater recruitment drives among underrepresented groups or more purposeful sampling to take the bias of the wider Flusurvey group into account.

A further limitation was that not all respiratory viruses were tested for (eg, coronavirus and enterovirus). However, because this was a proof-of-concept study for Flusurvey, a crowd-sourcing platform for influenza surveillance, the focus remained on assessing influenza and other respiratory infections were considered to be supplementary. This may account for the individuals who reported symptoms, but whose swabs tested negative for influenza.

### Conclusion

This pilot study has shown that it is feasible for individuals to conduct self-swabbing for ILI/ARI in their own home at relatively low cost. Those selected to participate were able to successfully collect samples and the biological material gathered was sufficient for influenza and other viruses to be detected in the laboratory. This allows us to conclude that, as a proof of concept, it is possible to use home swabbing for detection of influenza at the community level. Due to the small sample size, conclusive statements about how effective the Flusurvey algorithms may be in comparison to other forms of community-based surveillance cannot be made, yet it still validates the conceptual approach used for online symptomatic surveillance methodology. However, there remain concerns about the accuracy of such a system and further research would be needed to repeat a similar experiment with a greater number of participants to provide a suitable sample size to make any broader assumptions about the accuracy of online influenza detection systems.

## Appendix

Multimedia Appendix 1 Information sheet for pilot participants.

Multimedia Appendix 2 Written instructions for self-swabbing.

Multimedia Appendix 3 Swab sample labels for laboratory testing.

Multimedia Appendix 4 Pilot evaluation form.

Multimedia Appendix 5 Anonymized dataset.

Multimedia Appendix 6 Additional dataset from secondary testing.

## Abbreviations

ARI: acute respiratory infection
GP: general practitioner
ILI: influenza-like illness
PCR: polymerase chain reaction
PHE: Public Health England
RCGP: Royal College of General Practitioners
