# Supplementary material for: Self-Swabbing for Virological Confirmation of Influenza-Like Illness Among an Internet-Based Cohort in the UK During the 2014-2015 Flu Season: Pilot Study
Source: J Med Internet Res. 2018 Mar 1;20(3):e71. doi: 10.2196/jmir.9084 (PMC5856931; doi:10.2196/jmir.9084)
Supplement: Multimedia Appendix 1 [file jmir_v20i3e71_app1.pdf]

January 2015

Dear Participant,

You have been randomly selected from those who confirmed that they would be willing to take part in the self-swabbing exercise as part of Flusurvey research this year.

<u>Please do not undertake the self-swab until we ask you to do so.</u> Please continue to fill in your weekly symptoms survey online each week (flusurvey.org.uk) when prompted by email. If you report flu-like symptoms we will contact you by email and ask you to undertake the self-swab exercise. In the meantime, please keep this pack in a safe place.

Included in this pack is all the content that you need to participate in this self-swabbing exercise. This includes:

- Information sheet on the study
- Instructions for how to undertake the self-swabbing
- Swab pack
- Sticker with your unique ID to place onto your swab tube
- Transport tube & lid
- Postal box
- Instructions for building the postal box
- Sticker to seal the postal box
- Letter for Public Health England to enclose with your sample

When instructed to do so by the Flusurvey team, please undertake the self-swabbing. Video guidance for how to do this can be found at **http://www.flusurvey.org.uk/en/virological-**

swabbing. Written instructions are also provided in this pack. Please swab your nose accordingly and then follow the instructions to send it back to the Public Health England laboratory. It's important that you remember to stick your unique identifier sticker onto the tube containing the swab (not the larger transport tube) and make sure that you enclose the letter to Public Health England in the return box. When completed, this should be returned to the laboratory with the pre-paid postal box.

If you have any questions during the process, or wish to give feedback, please do not hesitate to contact clare.wenham@lshtm.ac.uk. We wish to take this opportunity to thank you for your time and effort in undertaking this Flusurvey self-swabbing study, and we look forward to sharing the results with you shortly.

Yours Faithfully

The Flusurvey Team

Virological Swabbing Participant Information Sheet

You have been invited to take part in an additional flusurvey sub-study. This year, in addition to collecting information provided by you online relating to your symptoms during the flu season, we are asking a small sample of people to undertake a swabbing exercise, so we can ascertain whether the symptoms that you may experience are caused by an influenza virus, or not.

Before you decide to take part it is important for you to understand why this research is being done, and what it will involve. Please take time to read the following information carefully and to talk to others about the study, if you wish. E-mail us (clare.wenham@lshtm.ac.uk) if there is anything that is not clear or if you would like more information.

What is the purpose of this study?

Flusurvey has been running for a number of years without any virological testing. This has meant that although the survey has been able to make estimates about the burden of influenza-like-illness in the UK, we cannot say anything about the burden of influenza (flu) itself. This year, we are piloting virological swabbing for the first time, to understand whether those people who report symptoms of influenza-like-illness through Flusurvey are actually suffering from influenza, or or some other infection.

Who is funding this research?

The Flusurvey project for virological swabbing has been funded by Engineering and Physical Sciences Research Council (ESPRC) as an I-sense exploratory project. I-sense is a broader project with a collaboration of institutions examining Early Warning Sensing Systems for Infectious Diseases (http://www.i-sense.org.uk).

Who are we selecting to take part and who is not able to take part?

We are selecting a subsample of the Flusurvey participants who are representative of the UK population. As we are trialing this as a proof of concept study and vaccinated individuals are less likely to be positive for influenza, if you have been vaccinated against flu in this current season (2014/5), we will not be able to include you in this part of the study. As this is a pilot study, we are only selecting 700 participants to take part.

What do we ask of you?

Individuals recruited into the study will be sent the self-sampling kits with instructions and asked to store them in a safe place until needed. Please do not use these kits until we contact you to do so. It's important that you undertake the self-swabbing whilst you are displaying symptoms of influenza-like-illness.

Please continue to fill in the Flusurvey reports online when prompted by email each week. If you begin to experience symptoms of influenza like illness, and report these to Flusurvey we will contact you and ask you to under take the self-swabbing exercise. *Please do not undertake the self-swabbing until we ask you to do this*. It is important that we get participants to administer the self-swab within two days of the date of the report to Flusurvey and during the estimated peak flu season 2014-15. This will be determined by PHE laboratory surveillance.

Video content showing how to administer the swabbing test will be available online (www.flusurvey.org.uk). Paper instructions of how to administer the self-swab will be provided with the swabs.

Once you have self-administered the swab, we ask you to return it to PHE laboratory in the pre-paid packaging included with your swabs. The packaging provided will ensure that the samples are protected whilst in the post so that they are secure and do not get damaged in transit. Please make sure that you seal the swabs in the packaging provided. This packaging will be compliant with UN3373 for the transport of biological samples.

You will also be provided with a Near Patient Test (subject to commercial partner donation). We would also ask you to administer this test separately. This rapid result test will provide you with instant results. We would ask that you email the results of this test to us at npt@flusurvey.org.uk. If possible, we would also like you to photograph this test result and attach this to the email. After this, you can dispose of this test in your home.

After you have completed both the tests, we will ask you to complete a questionnaire online, about your experience of the self-administered swabs and the acceptability of both tests. We ask that you be honest with your answers as it is useful for us to know how you found using both of these tests.

Why do we need your address?

We need your address so that we can send you a swab kit and return packaging so that you can administer the self-swabbing in your own home. These personal details will be stored at London School of Hygiene and Tropical Medicine for the duration of the flu season 2014/5 and then will be destroyed. Your personal details will not be shared with any third party during this time and will not be used for any purpose beyond the scope of this study.

The swab kit will not contain any personal details, and will be identified only by a unique number. This means that the PHE laboratory will not have any personal identifying details on the swabs received.

What will happen to my swab sample?

Swabs will be sent to Bristol PHE laboratories. Using multiplex-PCR, PHE will use standard diagnostic set of influenza A and B, RSV, Parainfluenza 1,2,3, adenovirus, human metapneumovirus, and rhinovirus. The primary outcome measure will be the yield (fraction of influenza positive tests received), and will be compared with that obtained by the GP-based virological sampling run by PHE in the same weeks. Testing for a broad panel of viruses will demonstrate the likely aetiology of non-flu respiratory illness. These swabs will contain a unique code that will be assigned to you by Flusurvey so that those at PHE will only receive anonymous samples. This is to ensure your privacy during this research. Please ensure that you enclose no personal identifiable information along with your sample.

Anonymised results from the virological swabbing will be returned to the Flusurvey team, de-anonymised and then the results will be e-mailed to you. You will therefore know what virus was the likely cause of your illness.

Once you have undertaken the Near Patient Test, and sent the results to us by email, you can dispose of the test in your own home. The results from this test will be used in a similar way to that of the main virological swabbing study and will be compared to the data obtained from the GP/hospital virological sampling run by PHE in the same weeks.

Please be advised – the results from these tests do not constitute medical advice. The results will only be able to confirm to participants whether they had an influenza virus present on their swab on that day. Due to the considerable number of swabs that are received by PHE laboratories on any one week, both from this study and those coming from GP and hospital

swabbing, there will be a delay in testing and returning of results. This may mean that the results of the testing are not returned to you until after your episode of flu like symptoms have passed. If you have any concerns about your health, please consult your GP.

After the tests have been carried out at PHE, the anonymised swab samples will be sent to University College London (UCL) laboratories, under the guidance of Prof Rachel McKendry for further characterisation of the virus and to aid in-house assay development. This project is funded under the same I-sense EPSRC / IRC funding as this exploratory project. These samples will then be destroyed before the end of the I-sense funding cycle (31st October 2018).

Are there any risks involved?

If the self swabbing is carried out as shown on the Flusurvey website and as included in the participant leaflet received with the swab, there should be no risks involved in undertaking the virological swabbing. Participants may experience discomfort when administering the self-swab, but this will only be momentary whilst undertaking the swabbing.

Do you have to take part? What if you change your mind?

Participation in this virological swabbing study is completely voluntary. Participants are asked to take part out of good will in order to improve our understanding of the burden of influenza in the UK. You must consent to the study before taking part in the swabbing exercise.

If, during the study, you change your mind about participation, let us know so we can withdraw your name and personal details from the database. You can withdraw from the study at any time, without disclosing the reason. If you withdraw from the study, we will destroy all identifiable samples, but we may need to use the data collected up to your withdrawal.

How will the data be used as part of the study?

The results derived from your tests will be anonymised and aggregated. This data will be used to understand how many people who report influenza like symptoms have a related virus detected on their swab. The number of positive cases of such viruses amongst the swabs received will be compared to the number of positive cases ascertained from the PHE laboratories collected through GP and hospital swabs for that week.

What will happen to the results of the research study?

We will use aggregated data of the results of the virological swabbing and near patient tests to assess how many participants who report influenza like symptoms are suffering from an influenza virus. We will use this data in preliminary findings on the Flusurvey website and in academic publications. You will not be identified in any report or publication, as the data analysed will be anonymous and aggregated.

What about my data protection and confidentiality? Who will see my data?

Your privacy is important to us and as such we will ensure that your data will be kept safe at all times. Only the team at Flusurvey will hold any personal information about you and the samples will have an anonymised code applied to them. That way, the laboratories at PHE and UCL will not know whose samples they are testing.

The team at Flusurvey will hold your personal details for sending out the swabs and returning test results only. Your details will not be used for any other reason. Flusurvey will have a duty of confidentiality to you as a research participant and nothing that could reveal your identity will be disclosed outside the research site.

Your data and samples will be used for the purpose of this study by Flusurvey and the associated characterization of the virus at UCL, and will not be used for any further purpose.

Any results or publications derived from the work in which your samples have been used will only refer to anonymised and aggregated data.

This research complies with the Data Protection Act 1998 and this study was given a favourable ethical opinion by the London School of Hygiene and Tropical Medicine Research Ethics Committee.

Who will be undertaking the research and will be handling the samples

Principal Investigator – John Edmunds, Dean of Faculty of Epidemiology and Population Health, London School of Hygiene and Tropical Medicine

Flu Survey Coordinator – Clare Wenham, Department of Infectious Disease Epidemiology, London School of Hygiene and Tropical Medicine

Virological testing will be carried out at Public Health England Laboratories, Bristol, under the guidance of Dr Matthew Donati and his team.

Further tests on any remaining sample material will be transferred securely to University College London laboratories, under the guidance of Prof Rachel McKendry for further characterization to aid in house assay development. The PHE & UCL teams will not receive any personal identifiable information.

Analysis of the anonymised data will be carried out by the Flusurvey team, Department of Infectious Disease Epidemiology, London School of Hygiene and Tropical Medicine

Further information

If you have any queries about this study, please contact Clare Wenham

clare.wenham@lshtm.ac.uk

Many thanks in advance for your time and help with this important research project.

You will be given a copy of the information sheet and a signed consent form to keep.

Thank you for considering taking the time to read this sheet.
