# Supplementary material for: Self-Swabbing for Virological Confirmation of Influenza-Like Illness Among an Internet-Based Cohort in the UK During the 2014-2015 Flu Season: Pilot Study
Source: J Med Internet Res. 2018 Mar 1;20(3):e71. doi: 10.2196/jmir.9084 (PMC5856931; doi:10.2196/jmir.9084)
Supplement: Multimedia Appendix 2 [file jmir_v20i3e71_app2.pdf]

i-sense

1. Please watch the Nasal Swab instructions video (flusurvey.org.uk/en/virological-swabbing). This paper instruction sheet provides the same information as the video.

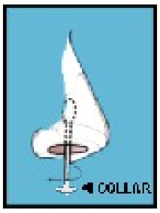

2. Take the sterile nasal swab out of its packaging. Carefully insert the dry swab 2.5 cm (or as far as you are comfortable) into one nostril and gently rotate the swab 3 times against the nasal wall.

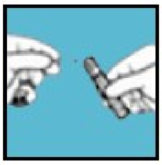

4. Break off the shaft of the swab and seal the tube tightly.

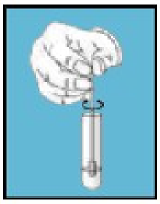

3. Immediately open the tube enclosed with the swab and put the nasal swab into it. Make sure you keep the red liquid in this tube as it preserves the sample until it reaches the lab.

5. Stick the sticker with your details on the small tube. This contains your participant ID so we can identify you.

6. Place this small tube, now sealed with the swab sample inside into the larger tube provided. Screw the lid provided onto this tube. This is to ensure that the samples are protected during transportation.

7. Please build the box to return the swab in. Please refer to the instructions provided with the box as to how to do this.

8. Please place the large tube, enclosing the smaller tube, into the box provided. Please enclose the sheet for Public Health England laboratory inside also. Seal the box with the plain sticker provided (or any tape if you prefer). The box has the address already written, and postage has been pre-paid. Please put this box into a post box as soon as you can.

With thanks to GoViral (ww.goviralstudy.com)

Any Questions: websmaster@flusurvey.org.uk
