# Supplementary material for: Self-Swabbing for Virological Confirmation of Influenza-Like Illness Among an Internet-Based Cohort in the UK During the 2014-2015 Flu Season: Pilot Study
Source: J Med Internet Res. 2018 Mar 1;20(3):e71. doi: 10.2196/jmir.9084 (PMC5856931; doi:10.2196/jmir.9084)
Supplement: Multimedia Appendix 3 [file jmir_v20i3e71_app3.pdf]

FLUSURVEY PILOT OF VIROLOGICAL SWABBING

PUBLIC HEALTH ENGLAND REFERENCE SHEET

Dear PHE Virology Lab.

Please find attached the nasal swab sample for participant:

UNIQUE PARTICIPANT IDENTIFIER: 001

POSTCODE: LL29 7RY

Please undertake PCR testing of this sample accordingly.

Any questions, please contact Clare Wenham, London School of Hygiene and Tropical Medicine, clare.wenham@lshtm.ac.uk

PLEASE REMEMBER TO STICK THE STICKER CONTAINING YOUR UNIQUE PARTICIPANT IDENTIFIER ONTO THE TUBE WITH THE SWAB ONCE UNDERTAKEN.

PLEASE INCLUDE THIS SHEET WITH THE SAMPLE IN THE BOX PROVIDED.
