# Supplementary material for: Self-Swabbing for Virological Confirmation of Influenza-Like Illness Among an Internet-Based Cohort in the UK During the 2014-2015 Flu Season: Pilot Study
Source: J Med Internet Res. 2018 Mar 1;20(3):e71. doi: 10.2196/jmir.9084 (PMC5856931; doi:10.2196/jmir.9084)
Supplement: Multimedia Appendix 4 [file jmir_v20i3e71_app4.pdf]

LONDON SCHOOL of HYGIENE &TROPICAL MEDICINE

Flusurvey Swabbing Evaluation

Page 1: Page 1

1 Did the swab kit arrive in good condition? * *Required*

1.a Please add any comments here:

2 How did you find undertaking the swab itself? * *Required*

2.a If you selected Other, please specify:

3 How easy was it to post the swab kits back to the laboratory? * *Required*

3.a If you selected Other, please specify:

4 Did you find the written instructions helpful? * *Required*

4.a Please let us know how we could improve these written instructions

5 Did you find the video instructions helpful? * *Required*

5.a. Please let us know how we could improve these written instructions

6: What could be done to improve the process of self-swabbing? * *Required*

7 Did you find the receipt of results easy to understand? * *Required*

7.a Please add any further comments here:

8: If you were to have flu like symptoms in the future, would you be interested in: * *Required*

8.a If you selected Other, please specify:

9 Why did you chose to participate in the Flusurvey Self-Swabbing pilot?

10 How do you find Flusurvey's system more generally? Please let us know if you have any comments on how we can improve the system for users

11 If you have any further comments about Flusurvey or the self-swabbing study, please let us know here.

Key for selection options

1 - Did the swab kit arrive in good condition?

Yes
No

2 - How did you find undertaking the swab itself?
Very Easy
Easy
Difficult
Very Difficult
Other

3 - How easy was it to post the swab kits back to the laboratory?
Very Easy
Easy
Difficult
Very Difficult
Other

4 - Did you find the written instructions helpful?
Yes
No
Did not use

5 - Did you find the video instructions helpful?
Yes
No
Did not use

7 - Did you find the receipt of results easy to understand?
Yes
No

8 - If you were to have flu like symptoms in the future, would you be interested in:
Undertaking a self-swab at home to diagnose symptoms
Visiting a GP
Neither of the above
Other
